# High endothelial venule is a prognostic immune‐related biomarker in patients with resected intrahepatic cholangiocarcinoma

**DOI:** 10.1111/cpr.13513

**Published:** 2023-07-03

**Authors:** Yan Zhou, Qian Gu, Linxi Zhu, Shuo Zhang, Hongyan Wu, Xiaohong Pu, Chunping Jiang, Jun Chen

**Affiliations:** ^1^ Department of Pathology, Nanjing Drum Tower Hospital The Affiliated Hospital of Nanjing University Medical School Nanjing China; ^2^ Department of Hepatobiliary Surgery Drum Tower Clinical College of Nanjing Medical University Nanjing China; ^3^ Department of Cardiology First Affiliated Hospital of Nanjing Medical University Nanjing China; ^4^ Department of Pancreatic surgery, Nanjing Drum Tower Hospital The Affiliated Hospital of Nanjing University Medical School Nanjing China; ^5^ Jinan Microecological Biomedicine Shandong Laboratory Shounuo City Light West Block Jinan City China

## Abstract

Having been reported to be a crucial prognostic factor in solid tumours, the role of high endothelial venule (HEV) in intrahepatic cholangiocarcinoma (ICC) remains unclear, however. The data of ICC and healthy individuals were downloaded from the Gene Expression Omnibus (GEO), and The Cancer Genome Atlas (TCGA) databases. Meanwhile, a cutting‐edge ICC high‐resolution spatial transcriptome was also acquired before these data were comprehensively analysed using bioinformatics approaches. Moreover, 95 individuals with ICC who had undergone resection surgery were enrolled in this study to investigate the relationship between HEV and tumour microenvironment (TME) applying immunohistochemistry and multiple immunofluorescence techniques. The high‐HEV subtype contains rich immune infiltrates including tertiary lymphoid structure (TLS), CD8+ T cells, and CD20+ B cells. Furthermore, HEV and TLS exhibited a strong relationship of spatial colocalization. Correlated with improved prognostic outcomes in ICC, the high‐HEV subtype could be an independent prognostic indicator for individuals with ICC. This study revealed the association of HEV with immune function and observed a strong spatial colocalization correlation between HEV and TLS. Moreover, correlated with immunotherapeutic response, HEV could improve prognostic outcomes, which may be a potential indicator of immunotherapy pathology in ICC.

## Graphical abstract

This study revealed the association of high endothelial venule (HEV) with immune function and observed a strong spatial colocalization correlation between HEV and tertiary lymphoid structure. Moreover, HEV was correlated with immunotherapeutic response and improved prognostic outcomes and may be a potential indicator of immunotherapy pathology in ICC.

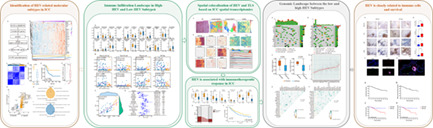

## 1 INTRODUCTION

Cholangiocarcinoma (CCA), a rarely occurring but aggressive malignant tumour, stems from the bile duct epithelium, which includes intrahepatic cholangiocarcinoma (ICC), perihilar cholangiocarcinoma, and distal cholangiocarcinoma as per its anatomical location.^1^ Over the past decades, ICC has gradually aroused public concern due to its rising morbidity and mortality worldwide as well as the heterogeneity of its histology and genetic alterations.^2^, ^3^ According to the fifth WHO Classification of Digestive System Tumours, ICC is categorized into small and large bile duct types. Despite recent advancements in cancer treatment, the prognosis of ICC remains bleak, with a <10% 5‐year overall survival (OS).^3^ While resection surgery stands as the only treatment option for individuals with ICC, only a portion of them are eligible to undergo this procedure because most patients diagnosed with advanced ICC are not candidates for surgical resection.^4^ The majority of the individuals with CCA take conventional cytotoxic chemotherapy as the main treatment option; however, compared with the best supportive care, it only extends their survival to a few months, along with certain adverse side effects.^5^ The limited effectiveness of CCA treatment is related to multiple factors. Although recent studies have pointed to immunotherapy as a treatment strategy for CCA,^6^ the comprehension of the tumour microenvironment (TME) remains limited.

From a histopathological standpoint, adaptive immune responses, including tumour‐infiltrating lymphocytes (TILs), along with their roles in controlling ICC growth and spread, are becoming increasingly evident.^7^ A declining number of TILs and/or their incompetency have been associated with poor survival outcomes and aggravated malignancy.^7^ According to a report on human ICC biopsies, TILs accounted for 8.0% of inflammatory cells in TME.^8^ Individuals with ICC featuring an increasing CD8^+^ T‐cell infiltration showed a higher OS and disease‐free survival and a lower risk of recurrence after radical tumour resection.^9^ Although CD20^+^ B lymphocytes are uncommon in ICC,^10^ their presence appears to indicate better OS in ICC patients.^11^

High endothelial venules (HEVs), a unique vessel type in secondary lymphoid organs such as lymph nodes, enables lymphocytes to exit from the systemic circulation and enter lymph nodes.^12^ Apart from the previously stated physiological roles of lymphoid organs, ectopic HEV can be observed in pathological conditions such as chronic inflammation and neoplasia.^13^, ^14^, ^15^, ^16^ Especially, intra‐tumoral high density of HEV has been recognized as a positive prognostic factor in numerous solid tumours, including malignant melanoma, head and neck cancer, and breast cancer,^16^, ^17^ and also has identified as a key determinant in lymphocyte trafficking.^18^ Moreover, intra‐tumoral HEVs could cooperate with lymphocytes to help them cross the endothelial barrier, according to an analysis of the coexistence of HEVs and lymphocytes in tumour tissue.^19^, ^20^ In addition, HEV is linked to other subtypes of T cells, such as memory T cells, B cells, and lymphotoxin‐producing dendritic cells (DCs),^20^ which could promote lymphocyte‐mediated tumour cell death.^16^, ^17^

Given the uncertainty of the role of HEV in ICC and its relationship with TILs, a comprehensive analysis was conducted in this research combining computational and laboratory methods to clarify these issues. This study also reveals the association of HEV and TILs as well as the effect of the former one on prognosis in resectable ICC patients.

## 2 MATERIALS AND METHODS

### 2.1 Patient samples

A total of 95 individuals with ICC who had undergone radical resection at Nanjing Drum Tower Hospital from 2017 to 2022 were enrolled in this study, in which those who had received neoadjuvant chemotherapy prior to surgery or had diagnosed with distant metastases were not included. Clinicopathological information of individuals at the time of diagnosis, including their age, gender, pathological stage, microvascular invasion, maximum tumour diameter, tissue type, tumour recurrence, and survival status, was retrieved from a prospectively maintained database with a pre‐designed format for data gathering.

### 2.2 Acquisition of transcriptomic data

Transcriptomic data and related clinicopathological data of 83 individuals with ICC were retrieved from the Gene Expression Omnibus (GEO) database (accession number: GSE89749). The same information for 30 individuals with ICC as well as 8 healthy samples were acquired from the Cancer Genomic Atlas (TCGA, https://portal.gdc.cancer.gov/). Moreover, the high‐resolution spatial transcriptomic data of ICC was retrieved from a study by Rui Wu et al. (http://lifeome.net/supp/livercancer-st/data.htm).

### 2.3 Differential expression analysis

The differential expression of mRNAs was assessed by means of the Wilcoxon rank sum test method. The screening criteria for selecting significant differentially expressed mRNAs were set as *p* < 0.05 and |log2 (fold change) | > 0.5.

### 2.4 Single‐sample gene set enrichment analysis

The single‐sample gene set enrichment analysis (ssGSEA) algorithm supported by the GSVA (v1.38.2) package in R was applied to calculate enrichment scores for individual samples. The differentially expressed genes (DEGs) obtained above were utilized as the gene set for ssGSEA.

### 2.5 Consensus clustering

The ConcensusClusterPlus (v1.60.0) package in R was applied to perform consensus clustering to establish the HEV‐associated molecular subtypes. First, the ideal cluster numbers between *k* = 2 and 9 were analysed, which was repeated 1000 times to ensure stable results. Then, the pheatmap (v1.0.12) package in R was employed to generate a cluster map.

### 2.6 Functional enrichment analysis

Kyoto Encyclopedia of Genes and Genomes (KEGG) and Gene Ontology (GO) analyses were performed to examine the differential physiological processes and signal pathways between the low‐HEV and high‐HEV cohorts. The mentioned analyses were conducted utilizing the clusterProfiler (v4.4.4) package. The *p*‐value threshold was set as 0.05. Besides, Radar plot was used to demonstrate the outcomes of KEGG and GO analyses.

### 2.7 Immune infiltration analysis between the low‐HEV and high‐HEV cohorts

To explore the immune features of ICC samples, TIMER v2.0 was applied to determine the relative fraction of 22 immune cell types, which then was compared between the low‐HEV and high‐HEV cohorts. The findings were illustrated in a landscape map.

### 2.8 Prediction of response to immunotherapy

Tumour immune dysfunction and exclusion (TIDE) analysis was utilized to explore immunotherapy response in cancer treatment. And it (http://tide.dfci.harvard.edu/) was also applied to predict immunotherapy response based on two major tumour immune evasion mechanisms: T‐cell dysfunction and reduced T‐cell infiltration.

### 2.9 Somatic mutation analysis

Somatic mutation data of the ICC samples were retrieved from the TCGA database in ‘maf’ format. The top 30 mutated genes were acquired and presented in the waterfall plot by means of the maftools package (v2.12.0) in R, which is usually applied to visualize and summarize the mutated genes.

### 2.10 Immunohistochemical staining

The fixation of ICC samples was first performed using 4% formaldehyde solution before they were embedded in paraffin. The formalin‐fixed paraffin‐embedded tissue specimens were cut into sections of 4 μm and incubated with MECA‐79 (Abcam, ab111710), CD8 (Abcam, ab237709), and CD20 (Abcam, ab78237) primary antibodies at 4°C (incubation time: 12 h). Subsequently, the sections were subjected to incubation at ambient temperature for 1 hour with the HPR‐labelled secondary antibodies. After that, the samples were subjected to 3,3'‐diaminobenzidine and haematoxylin staining. The intensity of staining was independently assessed by two experienced pathologists. TME patterns were also independently evaluated based on 20 randomly selected regions of each slide by two experienced pathologists. The Ethics Committee of Nanjing Drum Tower Hospital (2016‐057‐01) approved this research, and all procedures were carried out following the Declaration of Helsinki and government policies. All subjects consented to participate in this study.

### 2.11 Survival analysis

The Kaplan–Meier (KM) curve was utilized to compare the OS across the low‐HEV and high‐HEV cohorts using the survminer and survival package in R. The prognostic indicators were detected by means of the univariate and multivariate Cox analyses.

### 2.12 Multiple immunofluorescence staining

Multiple immunofluorescence (mIF) was performed on a series of 5.0 μm histological tumour sections from a representative high‐HEV sample. These sections were treated using an opal fIHC kit (PerkinElmer, USA). Across all sections, the procedure of antibody staining was consistent. The sections were subjected to back staining with DAPI (Vector Laboratories, Germany). Multiple immunofluorescent plates include CD8, CD20, and MECA‐79. An antibody thinner (Dako, Germany) was utilized to dilute all antibodies. The secondary antibody was processed with the ImmPRESS™ HRP (peroxidase) polymer Cancer Detection kit (Vector Laboratories, USA). A 1 × Plus amplification thinner (PerkinElmer/Akoya Biosciences, 01752, USA) was utilized to dilute the TSA reagent.

### 2.13 Statistics

Statistical analyses were conducted in R software (v4.2.0). Spearman correlation was utilized to determine the correlations across immune cell subsets. Survival analysis was measured using the log‐rank test. The Benjamini–Hochberg method was utilized to adjust the *p‐*value, in which *p* < 0.05 indicated a significance level.

## 3 RESULTS

### 3.1 Identification of HEV‐related molecular subtypes in ICC

**FIGURE 1:**
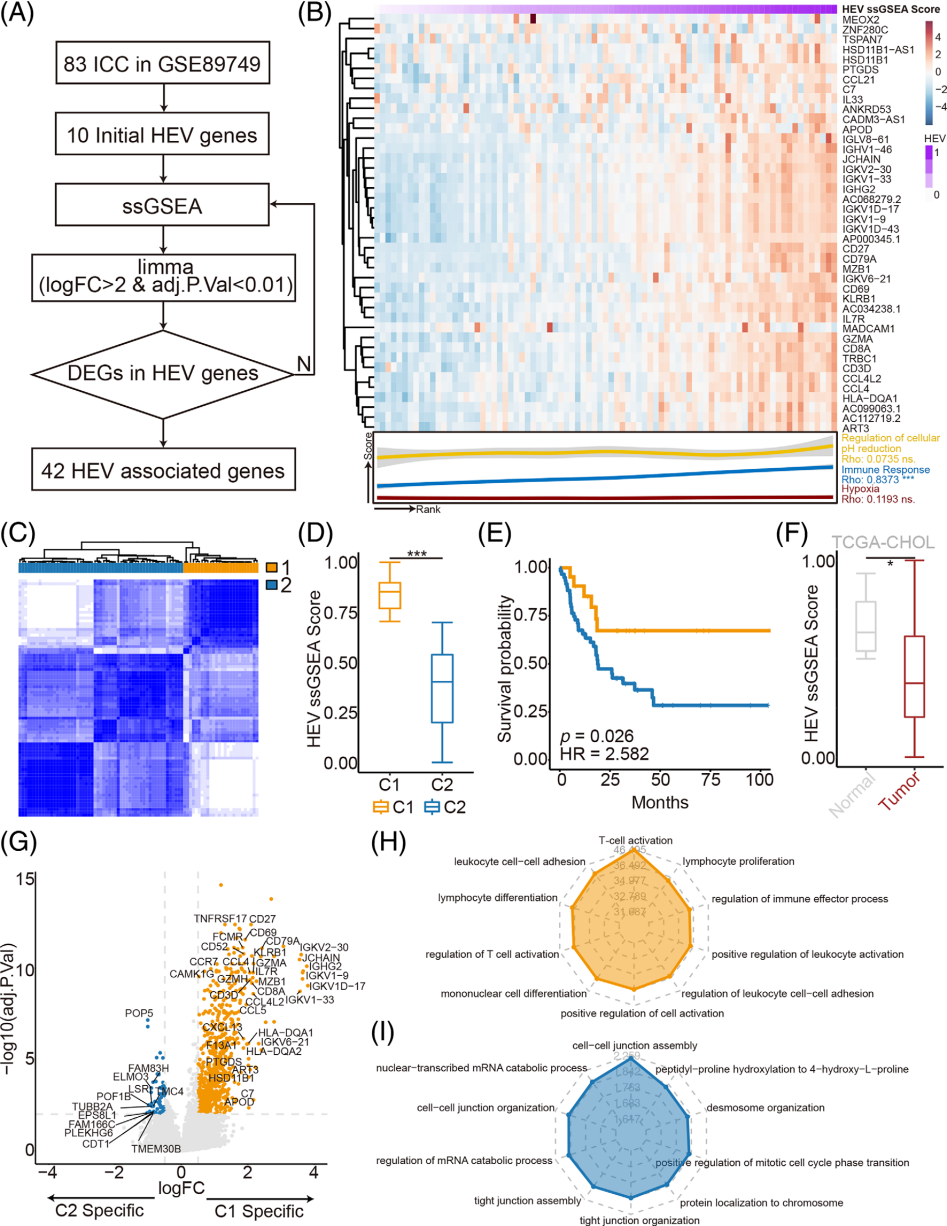
Identification of high endothelial venule (HEV)‐related molecular subtypes in intrahepatic cholangiocarcinoma. (A) The workflow of identifying key HEV‐related genes. (B) Heatmap highlighted the expression of HEV‐related genes in samples sorted by HEV single‐sample gene set enrichment analysis (ssGSEA) scores and the graph below shows the correlation between tumour environment and HEV ssGSEA scores. Colour scale: blue denotes low expression, red denotes high expression, and purple denotes HEV ssGSEA score. Red line: Hypoxia, blue line: Immune Response, and yellow line: Regulation of cellular pH reduction. (C) Consensus clustering based on HEV‐related genes. Orange: C1 with high HEV score; blue: C2 with low HEV score. (D) Box plots showed the HEV ssGSEA score of the two clusters. (E) Survival analysis of the patients grouped by C1 and C2 in GSE89749. (F) Box plots showed the HEV ssGSEA scores in tumour and normal samples in the TCGA‐CHOL dataset. (G) Differential expressing genes analysis between C1 and C2. (H,I) Top 10 GO terms enriched in the C1 (orange) and C2 (blue). P values were computed by a two‐sided Wilcoxon test. *p < 0.05, **p < 0.01, ***p < 0.001. DEGs, differentially expressed genes; HR, Hazard ratio.

In order to investigate the role of HEV in ICC, the ICC based on HEV‐associated genes was reclassified, and the potential distinction among different subtypes was explored. As illustrated in the flowchart (Figure 1A), 83 individuals with ICC were obtained from GSE89749 and classified into the high‐HEV and low‐HEV groups as per the ssGSEA scores from the ssGSEA algorithm, which was based on 10 HEV genes obtained by literature search (CHST4, CCL21, CCL19, IL33, ICAM1, MADCAM1, TSPAN7, MEOX2, ANKRD53, and ZNF280C). A total of 42 stable and specific HEV‐associated genes were retrieved through multiple iterations of differential expression analysis. Afterward, the 83 ICC samples were ranked according to the ssGSEA scores based on the 42 HEV‐associated genes and the expression levels of the 42 HEV‐related genes were presented using a heatmap (Figure 1B).

Numerous research reports have demonstrated that many microenvironmental factors orchestrate tumour progression. In order to identify the potential impact of HEVs on the TME, the correlation between HEV ssGSEA scores and multiple microenvironment‐related pathways was further tested, which include hypoxia, immune responses, and the regulation of cellular pH. Amid them, the immune response showed a considerably positive link to HEV ssGSEA scores (Spearman's correlation test, ρ = 0.837, *p* < 0.001, Figure 1B), suggesting the role of HEVs in the TME.

Afterward, the HEV‐associated clusters of ICC samples were determined by means of the consensus clustering method. Two clusters in the GSE89749 cohort were established with distinct HEV gene expression patterns after *k*‐means clustering (Figure 1C). Moreover, cluster C1 showed a high HEV ssGSEA score, indicating that it was the high‐HEV subtype, whereas cluster C2 presented a low HEV ssGSEA score, suggesting that it was the low‐HEV subtype (Figure 1D). Furthermore, survival analysis illustrated that the high‐HEV subtype had a favourable prognosis in contrast to the low‐HEV subtype (Figure 1E). In addition, HEV ssGSEA scores in the TCGA‐CHOL cohort were assessed, and it was observed that HEV ssGSEA scores were down‐regulated in the tumour samples in comparison to the normal samples (Figure 1F).

To comprehend the underlying molecular mechanism of HEV in ICC, a differential expression analysis and a gene functional enrichment analysis based on DEGs were performed. The high‐HEV subtype showed a dominant immune‐associated phenotype, with the high expression of CD79A, CCR7, IL7R, CD3D, IGHG2, and CD8A (Figure 1G). Moreover, the up‐regulated genes in the high‐HEV subtype were enriched in biological processes correlated with immunity, such as T‐cell activation, leukocyte cell–cell adhesion, lymphocyte differentiation, regulation of T‐cell activation, and mononuclear cell differentiation pathways (1H). In contrast, that in the low‐HEV subtype were enriched in cell–cell junction assembly, nuclear‐transcribed mRNA catabolic process, cell–cell junction organization, and regulation of mRNA catabolic process pathways (Figure 1I). These outcomes highlighted that the high‐HEV subtype was linked to the immune‐active microenvironment.

### 3.2 Immune infiltration landscape in high‐HEV and low‐HEV subtypes

**FIGURE 2:**
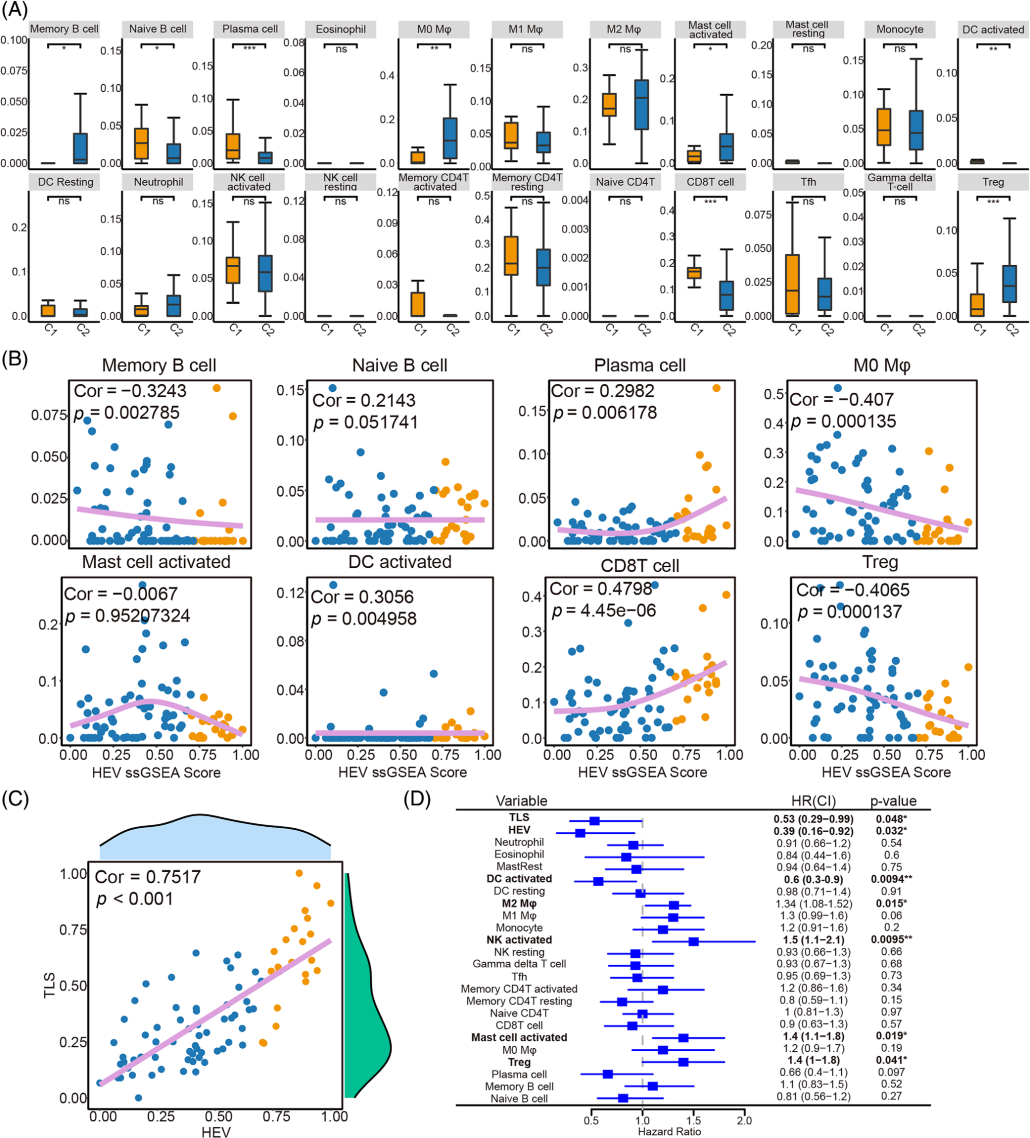
Immune infiltration landscape in high‐HEV (high endothelial venule) and low‐HEV subtypes. (A) Immune infiltration of 22 immune cell types of the C1 and C2 groups by cibersort. (B) Correlation analysis between immune cell types and HEV signature. (C) Correlation analysis between tertiary lymphoid structure signature and HEV signature. (D) Univariate Cox analysis of the 24 immune cell types in GSE89749. Correlation analysis calculated with Spearman's Rank correlation coefficient. *p < 0.05, **p < 0.01, ***p < 0.001. ssGSEA, single‐sample gene set enrichment analysis; HR, Hazard ratio.

Evidence suggests that HEVs are essential for the function of the immune system. In this research, the differences (variations) in immune infiltration of 22 kinds of immune cells in the TME between the high‐HEV subtype (C1) and the low‐HEV subtype (C2) were assessed based on the ICC cohort from GSE89749. The result showed that the high‐HEV subtype exhibited remarkably elevated percentages of naïve B cells, plasma cells, activated DCs, and CD8 T cells as well as substantially reduced proportions of memory B cells, M0 macrophages, activated mast cells, and Treg cells (Figure 2A). Naïve B cells, plasma cells, activated DCs, and CD8 T cells showed significant positive associations with the HEV ssGSEA score. Memory B cells, M0 macrophages, activated mast cells, and Treg cells showed a clear opposite trend in the correlation analysis (Figure 2B). These findings revealed that the high‐HEV subtype was correlated with the immune‐hot phenotype, whereas the low‐HEV subtype was correlated with the immune‐cold phenotype.

Tertiary lymphoid structures (TLSs), ectopic lymphoid structures composed predominantly of B cells, T cells, and DCs, have been strongly associated with improved survival and clinical outcome upon cancer immunotherapies. Consistent with the immune infiltration results mentioned above, the HEV ssGSEA scores also showed a high positive correlation with the TLS ssGSEA scores (Spearman's correlation test, ρ = 0.7517, *p* < 0.001; Figure 2C), implying that TLS might surround HEVs. Subsequently, a prognostic model based on HEV ssGSEA scores, TLS ssGSEA scores, and 22 immune cell infiltration scores was constructed. HEV, TLS, and activated DCs were observed to be substantially associated with the OS of individuals with ICC, as per the univariate Cox analysis results. Moreover, the HEV had a lower HR value, indicating its role as a good prognostic indicator for ICC samples in survival analysis (Figure 2D).

### 3.3 Spatial colocalization of HEV and TLS based on ICC spatial transcriptomics

**FIGURE 3:**
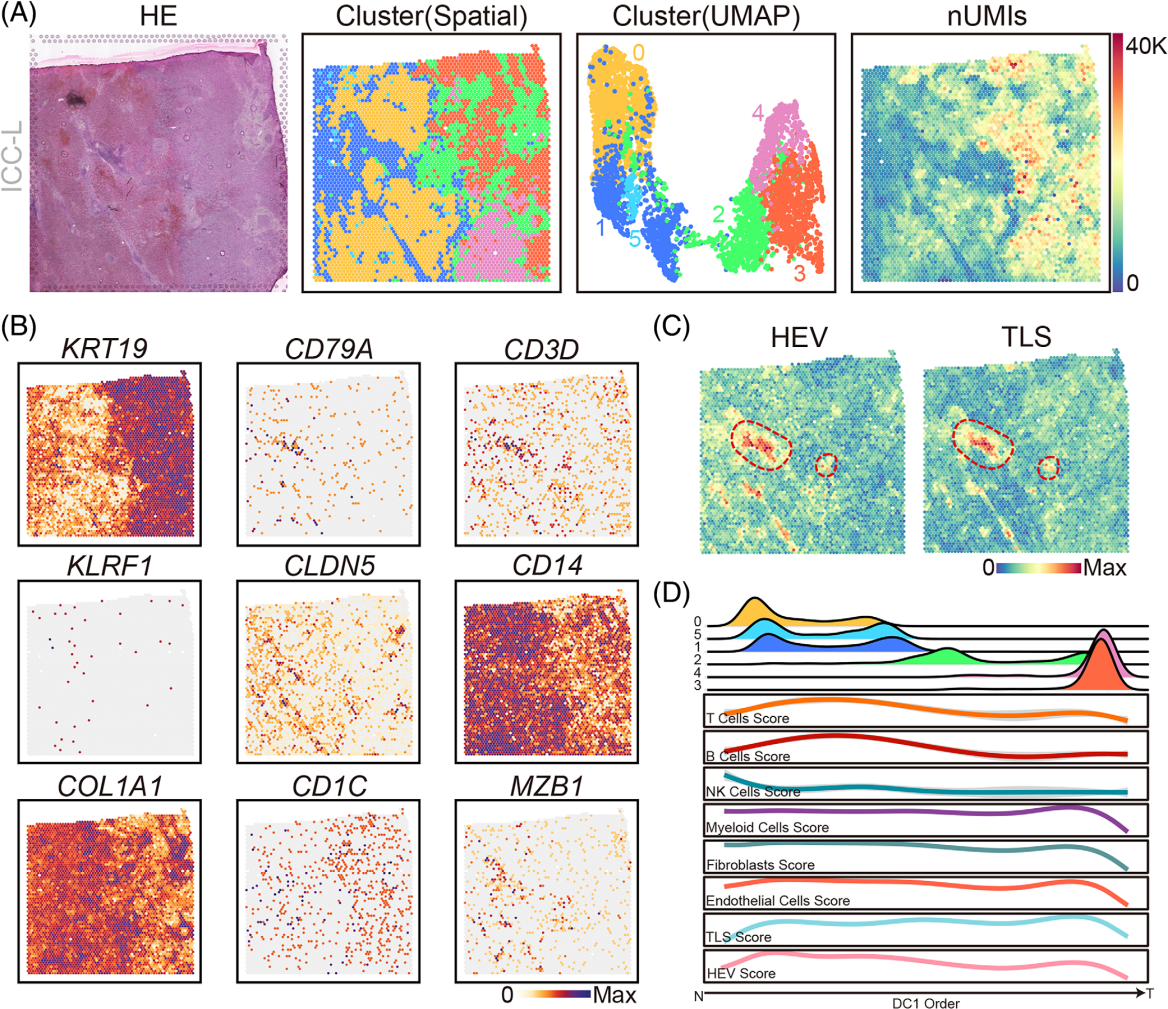
Spatial colocalization of high endothelial venule (HEV) and tertiary lymphoid structure (TLS) based on intrahepatic cholangiocarcinoma spatial transcriptomics (ST). (A) Hematoxylin and eosin staining (left), the spatial cluster distribution and the UMAP of the cluster distribution (middle), and the unique molecular identifiers (UMIs) number (right) of the spot of ST sections. (B) Uniform Manifold Approximation and Projection (UMAP) plot of key marker genes expression from the sections. The colour shade highlights the expression of the corresponding gene. (C) UMAP plot of HEV (left) and HEV (right) enrichment scores from the sections. Each dot denotes a spot of ST sections, and the colour shade highlights the scores of the corresponding type of cell. The red dashed circles represent the co‐location situation. (D) Distribution of the clusters (top plot of each cluster; *y*‐axis, the cluster's density) and the main stromal and immune cell type scores (bottom plot; *y*‐axis, the fitted cell type enrichment score) along the direction of the first diffusion map component (DC1 order, the shared *x*‐axis).

In order to better reveal the co‐occurrence of HEV and TLS, one piece of leading‐edge ICC spatial transcriptomics data from a study by Rui Wu et al. was re‐analysed. Characterizing the spatial diversity of the ICC samples, clustering analysis was performed, and the distribution of the clusters was revealed in the UMAP (Uniform Manifold Approximation and Projection) projection space as well as physical tissue space (Figure 3A). Having been extensively reported in the literature as a specific marker for malignant cells in ICC, KRT19 was highly expressed in the right regions of the pathological image, indicating that these regions were affected by cholangiocarcinoma (Figure 3B). Furthermore, the numbers of unique molecular identifiers (UMIs) in tumour areas were larger than that in healthy areas (Figure 3A). Moreover, the expression distribution of markers of essential stromal cells and immune cells was evaluated. The expression of CD14 (monocyte and macrophage marker) showed opposite trends from the expression of KRT19 (malignant cells marker), suggesting that monocytes or macrophages were enriched in normal regions compared with tumour regions.

Moreover, the expression of CD79A (B‐cell marker), CD3D (T‐cell marker), and MZB1 (Plasma cells marker) showed regional consistency with essential molecules for TLS formation (Figure 3B). As each spot contained several cells, the HEV and TLS enrichment level of various cell types was assessed in each spot based on the mean expression of specific cell markers. Likewise, the enrichment level of HEV and TLS in each spot was assessed based on the same strategy. With strong spatial colocalization, HEV and TLS were less enriched in tumour regions compared with normal regions (Figure 3C). Afterward, in order to assess the alterations in the composition of cells from the outside to the inside of the tumour, a diffusion map algorithm was utilized to project the spots into a 1S pseudo‐order (the first diffusion component, DC1), which is generally visible from healthy to tumour regions by the comparison of cluster distribution in the order explained. By fitting the variation curves of six types of cells, HEV and TLS scores, it was observed that the clusters related to the stromal regions shaped the variation patterns of fibroblasts, endothelial cells (EC), T cells, B cells, and even HEV and TLS (Figure 3D).

### 3.4 HEV is associated with immunotherapeutic response in ICC

**FIGURE 4:**
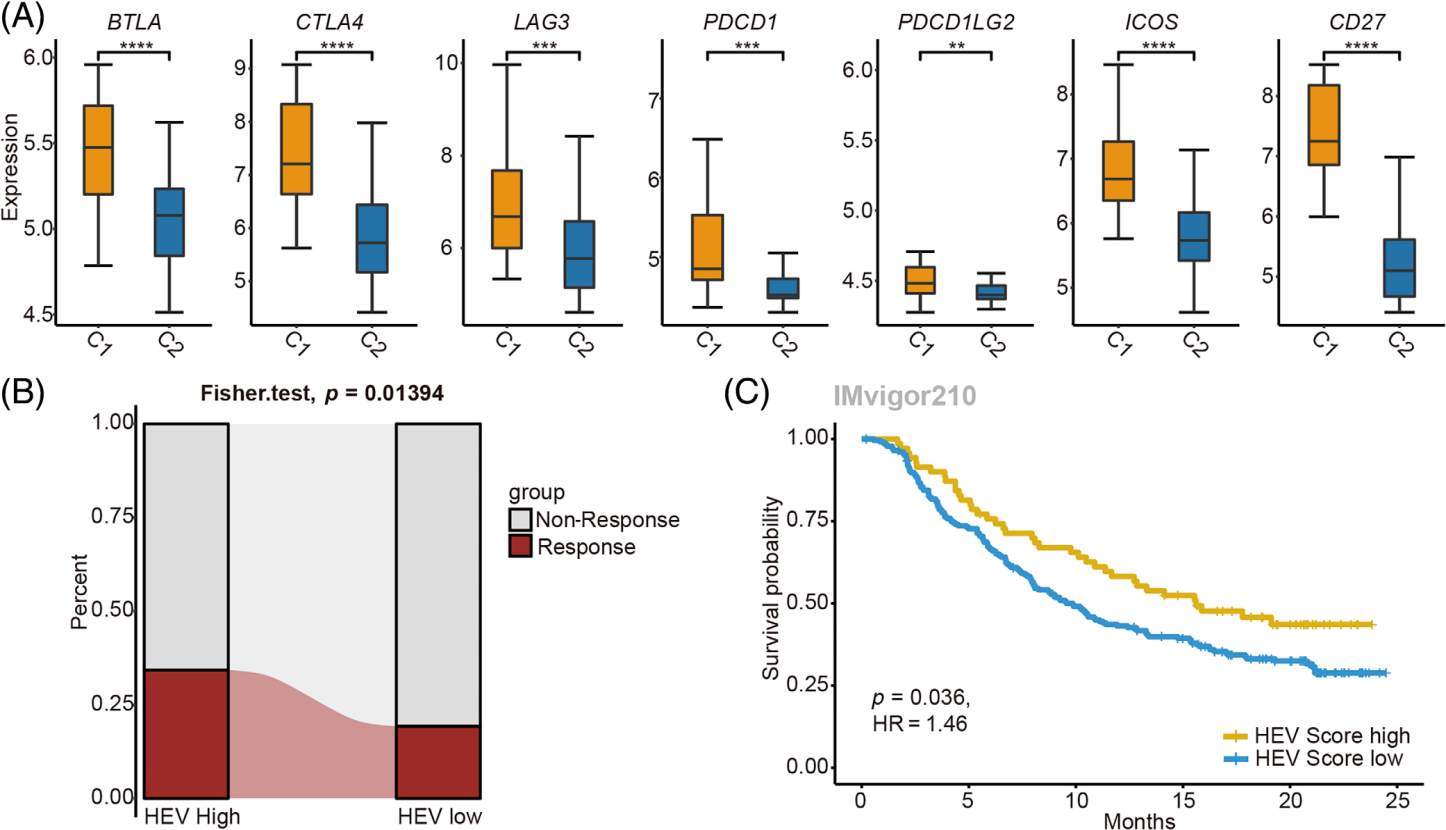
High endothelial venule (HEV) is associated with immunotherapeutic response in intrahepatic cholangiocarcinoma. (A) Box plots show the expression of several immune checkpoints between C1 and C2 groups (Wilcoxon rank sum test, *p < 0.05, **p < 0.01, ***p < 0.001, ****p < 0.0001). (B) The distribution of patients in the immunotherapy‐response group between HEV high and low groups in the IMvigor210 cohort (Fisher's exact test, *p* = 0.01394). (C) Survival analysis of the patients grouped by HEV signature in the IMvigor210 cohort.

In order to examine the relationship between HEV and immunotherapeutic effects, the expression profiles of immune checkpoints were assessed between the low‐HEV and high‐HEV subtypes. The results showed that several immune checkpoints were up‐regulated in the high‐HEV subtype in comparison with the low‐HEV one (Figure 4A). Subsequently, the TIDE algorithm was applied to assess the response of patients to immunotherapy. The high‐HEV group had a higher response rate than the low‐HEV group (Figure 4B), indicating the association of HEV with immunotherapeutic effects. Furthermore, the association between the HEV and prognostic outcomes of cancer patients was investigated based on the IMvigor210 cohort who received Atezolizumab treatment. The results demonstrated that the high‐HEV patients exhibited better OS compared to those with low HEV (Figure 4C), further underscoring the association between HEV and immunotherapeutic response.

### 3.5 Genomic landscape between the low‐HEV and high‐HEV subtypes

**FIGURE 5:**
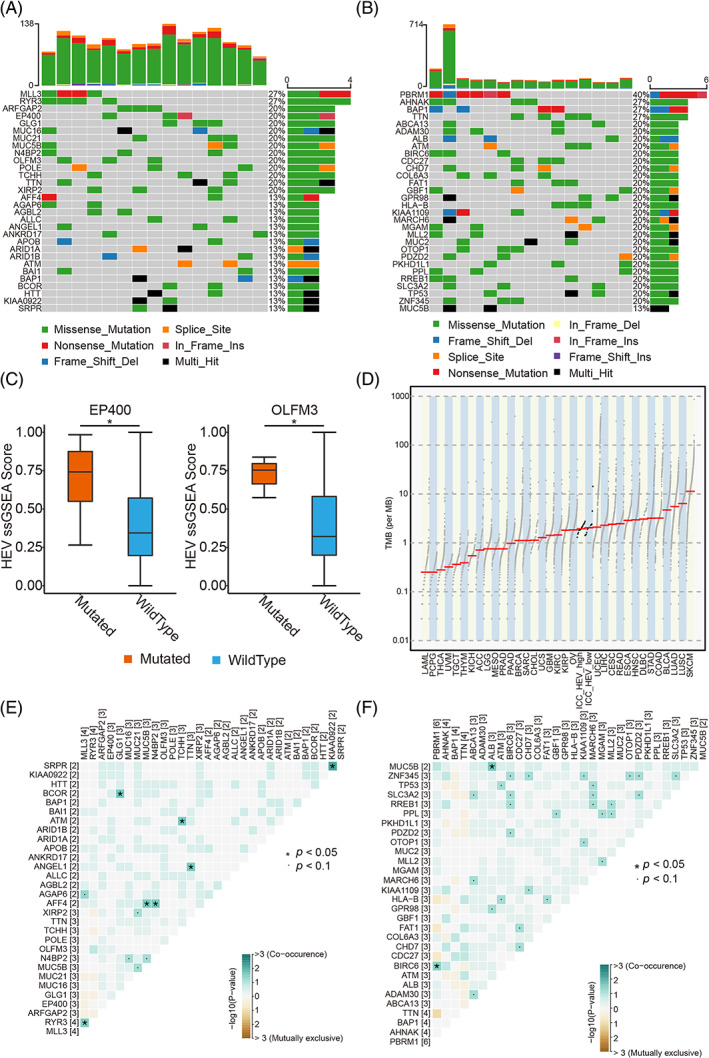
Genomic landscape between the low‐HEV (high endothelial venule) and high‐HEV subtypes. (A,B) Comparison of the variation in the mutation status of the top 30 genes with mutations between high HEV (A) and low HEV (B). (C) Box plots show that EP400 (left) and OLFM3 (right) gene mutations were considerably linked to high HEV signature (Wilcoxon rank sum test, *p < 0.05). (D) The TMB distribution of pan‐cancer in The Cancer Genome Atlas database. (E,F) Concurrence (blue) and mutual exclusion (brown) between high‐frequency mutation genes (the top 30 genes with mutations) in High‐HEV (E) and that in Low‐HEV (F) groups.

In order to determine the genomic landscape related to the HEV subtype, variations in the tumour‐intrinsic mutations between the low‐HEV and high‐HEV groups were compared in the TCGA‐CHOL ICC cohort. The results show that the group with high HEV had higher mutation rates of MLL3, RYR3, EP400, ARFGAP2, and GLG1 than that with low HEV (Figure 5A,B). In addition, the HEV ssGSEA score was considerably elevated in the group with mutated EP400 in comparison to the wild‐type group, and the HEV ssGSEA score was higher in groups with mutated OLFM3 than the groups with wild types (*p* < 0.05; Figure 5C). Moreover, the low‐HEV subtype had higher tumour mutational burden (TMB) levels than the high‐HEV subtype (Figure 5D).

The co‐occurrence and mutual exclusion between high‐frequency mutant genes were explored in this research, and it was revealed that RYR3 mutation was concurrent with MLL3 mutation in the high‐HEV subtype. Moreover, the AFF4 mutation was concurrent with MUC5B or N4BP2 mutation (Figure 5E). However, in the low‐HEV subtype, the BIRC6 mutation was mutually concurrent with the PBRM1 mutation, and MUC5B mutation was mutually concurrent with ALB mutation (Figure 5F).

### 3.6 Validation of the colocalization of HEV and immune cells

**FIGURE 6:**
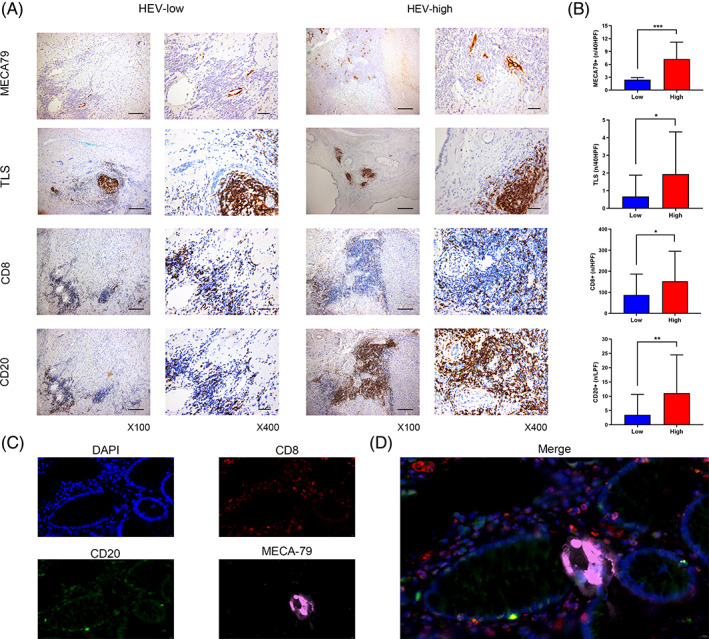
High endothelial venule (HEV) is co‐localized with immune cells. (A) The expression of HEV in intrahepatic cholangiocarcinoma (ICC) tissues was detected by MECA‐79 staining, and the ICC tissues were classified into low‐expression and high‐expression groups as per the median of HEV count (3/HPF). Anti‐CD8 and anti‐CD20 antibodies were utilized to stain the lymphocytes to evaluate their association with HEV density. Scale bar: 200 μm. (B) MECA‐79, CD8+, and CD20+ regions were quantified in the low‐HEV and high‐HEV groups, and their significant correlation was shown. (C) The blue box shows DAPI, the red box shows CD8, the green box shows CD20, and the pink box shows MECA‐79. (D) Images of four stains fused together, indicating spatial colocalization relationship between HEV and immune cells.

In order to validate the association of HEV and immune cells in ICC, 95 individuals with ICC who could undergo a resection surgery were selected from our hospital, and the HEV density of their surgically removed tumour sections was assessed. Based on the median HEV count (3/HPF), the tumour sections were classified into the low‐HEV (*n* = 60) and the high‐HEV groups (*n* = 30). It was observed that MECA79, TLS, CD8, and CD20 were substantially up‐regulated in the group with high HEV compared to the low‐HEV group (Figure 6A,B), and CD8+ or CD20+ lymphocytes clustered more near MECA‐79‐positive HEV in the high‐HEV group (Figure 6A,B). Subsequently, representative samples were selected in the high‐HEV group for mIFs staining to identify the correlation between HEV and immune cells. The results showed a prominent colocalization relationship between HEV and immune cells (Figure 6C,D).

### 3.7 HEV as an independent prognostic indicator in ICC

**FIGURE 7:**
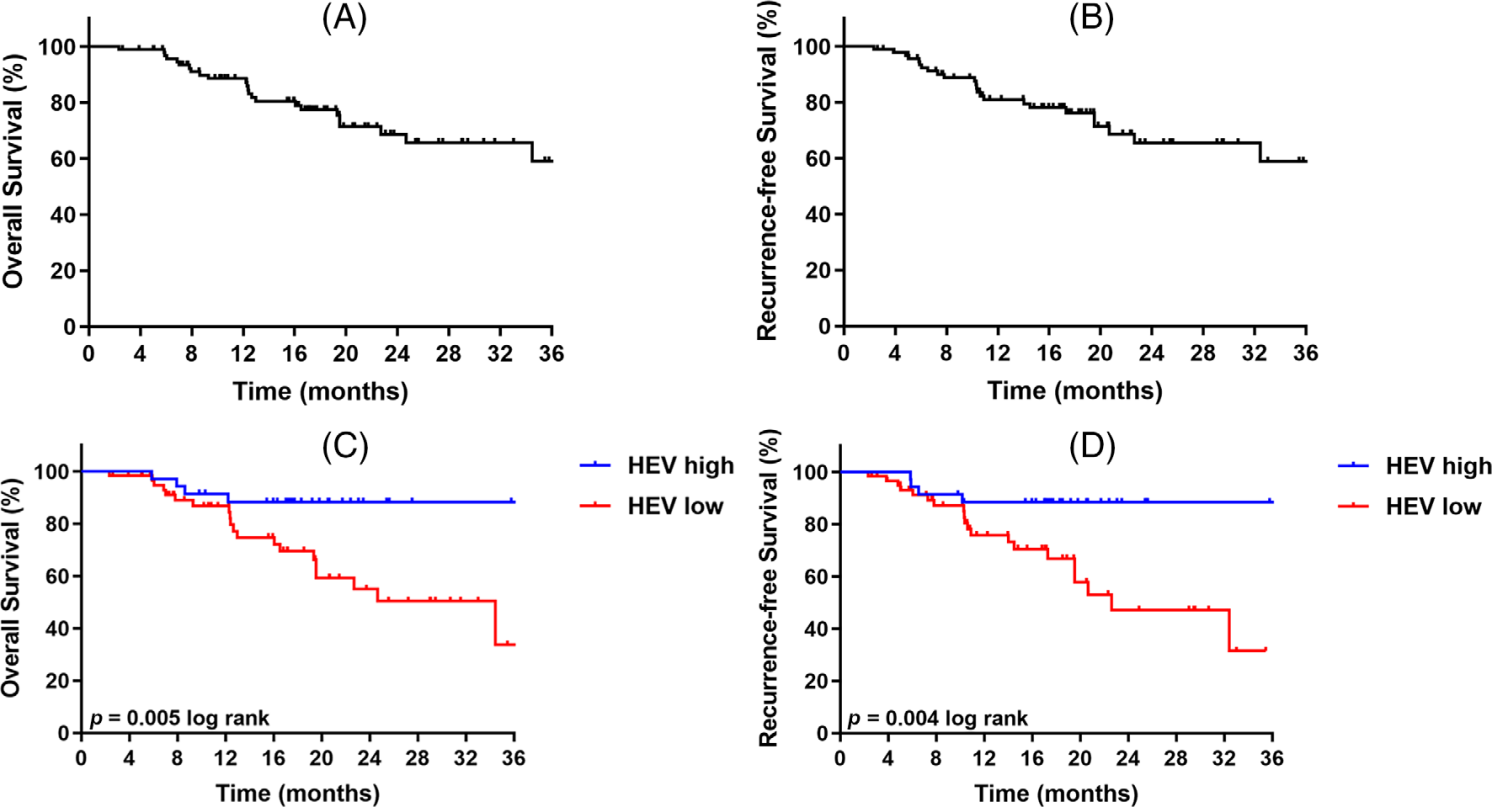
High endothelial venule (HEV) expression was associated with the survival of intrahepatic cholangiocarcinoma. (A) Overall survival. The median OS of the whole group was 20 months. (B) Recurrence‐free survival (RFS). The median RFS was 18 months. (C) Overall survival stratified by HEV. (D) RFS stratified according to HEV.

After the effect of HEV on the survival of individuals with ICC was analysed, KM analysis showed that HEV status significantly affected survival outcomes. Specifically, patients with high HEV had significantly longer RFS (*p* = 0.004 log‐rank) than those with low HEV. In addition, high HEV tumour is closely associated with prolonged OS (*p* = 0.005 log‐rank; Figure 7A–D).

In a further clinicopathological analysis (Table 1), the high‐HEV group was significantly associated with a smaller tumour diameter (*p* = 0.011). Concerning the tissue type, more HEVs were observed in the small bile duct type of ICC (*p* = 0.024). Based on immune cell composition and spatial structure in TME, immune cell infiltration was classified into ‘Desertic’ (a lack of immune infiltration of T and B cells), ‘Excluded’ (immune cells present at the tumour margins without the ability to penetrate the tumour bed), ‘Dispersed’ (immune cells infiltrate the entire tumour, but do not form an organized network) and ‘Structured’ (immune cells form TLS, which coordinate activation, maturation, and intra‐tumoral distribution of immune cells; Figure S1A). The high‐HEV group was found to be significantly correlated with the subtypes with rich immune cell infiltration (*p* < 0.001; Figure S1B). A further analysis of the Immunohistochemical (IHC) and prognostic data showed that more CD8^+^ (*p* = 0.002), TLS (*p* = 0.012), and CD20^+^ (*p* = 0.004) immune cells or structures infiltrated in the group with high HEV, which was considerably correlated with better DFS (*p* = 0.048) and OS (*p* = 0.003).

**TABLE 1:** Comparative analysis of surgically treated patients with respect to HEV.

Variables	HEV group		*p‐*Value
	High (*n* = 35)	Low (*n* = 60)	
Demographics			
Gender, *n* (%)			0.946
Male	16.0 (45.7)	27.0 (45.0)	
Female	19.0 (54.3)	33.0 (55.0)	
Age (years)	64.0 (53.0, 67.0)	63.0 (53.0, 68.8)	0.758
Pathological examination			
T category, *n* (%)			0.935
T1	20.0 (62.5)	34 (65.4)	
T2	11.0 (34.4)	16 (30.8)	
T3	1.0 (3.1)	2 (3.8)	
T4	0	0	
*N* category, *n* (%)			0.670
Nx	14.0 (43.8)	19.0 (36.5)	
N0	14.0 (43.8)	23.0 (44.2)	
N1	4.0 (12.5)	10.0 (19.2)	
Tumour grading, *n* (%)			0.206
G1	21.0 (61.8)	26.0 (44.8)	
G2	8.0 (23.5)	15.0 (25.9)	
G3	5.0 (14.7)	17.0 (29.3)	
G4	0	0	
Microvascular invasion, *n* (%)			0.717
M0	26.0 (81.3)	43.0 (84.3)	
M1	6.0 (18.8)	8.0 (15.7)	
Maximum tumour diameter (cm)	4.0 (3.1, 6.0)	6.0 (3.9, 7.9)	**0.011**
Histologic classification, *n* (%)			**0.024**
Small duct	23.0 (65.7)	25.0 (41.7)	
Large duct	12.0 (34.3)	35.0 (58.3)	
TME pattern, *n* (%)			**<0.001**
Desertic	8.0 (22.9)	43.0 (71.7)	
Excluded	16.0 (45.7)	12.0 (20.0)	
Dispersed	4.0 (11.4)	2.0 (3.3)	
Structured	7.0 (20.0)	3.0 (5.0)	
Immunohistochemical data			
CD8+, *n*/HPF	120.0 (120.0, 404.0)	55.0 (21.3, 107.5)	**0.022**
TLS, *n*/40 × HPF	1.0 (0, 3.0)	0 (0, 1.0)	**0.012**
CD20+, *n*/LPF	6.0 (1.0, 12.0)	1.5 (0, 4.8)	**0.004**
Follow‐up data			
Recurrence‐free survival (months)	19.2 (16.0, 25.5)	13.1 (7.4, 19.5)	**0.048**
Overall survival (months)	19.8 (16.0, 25.6)	15.8 (7.7, 21.3)	**0.003**

*Note*: Desertic: with an absence of T‐cell and B‐cell immune infiltration; Excluded: in which immune cells are present in the tumour margins but cannot penetrate the tumour bed; Dispersed: in which immune cells infiltrate throughout the tumour but cannot form organized networks; Structured: when immune cells form TLS that orchestrate the activation, maturation and intra‐tumoural distribution of immune cells. Bold indicates *p* < 0.05.

Abbreviations: HEV, high endothelial venule; HPF, high power field; LPF, low power field; TLS, tertiary lymphoid structures; TME, tumour microenvironment.

Further, to determine the potential of HEV as an independent prognostic indicator in ICC, a univariate and multivariate Cox regression analysis was conducted. In univariate analysis, HEV (Hazard ratio [HR] = 0.24; *p* = 0.009) and tumour grade (HR = 3.29; *p* = 0.006) were significantly correlated with OS (Table 2). All variables with *p* < 0.05 were imported into the multivariate Cox regression model. High HEV (HR = 0.28; *p* = 0.022) and tumour grade (HR = 2.50; *p* = 0.037) were determined as independent predictors of improved OS (Table 2). In simulated univariate analysis, high HEV (HR = 0.23; *p* = 0.007), tumour grade (HR = 3.17; *p* = 0.008), and small bile duct typing (HR = 2.31; *p* = 0.049) were considerably associated with RFS. In the multivariate Cox regression model, high HEV (HR = 0.31; *p* = 0.042) and tumour grade (HR = 2.67; *p* = 0.028) were deemed as independent predictors of RFS (Table 3).

**TABLE 2:** Univariate and multivariate analysis of overall survival.

Variables	Univariate analysis		Multivariate analysis	
	HR (95% CI)	*p*‐Value	HR (95% CI)	*p*‐Value
HEV (low = 1)	0.24 (0.08–0.70)	**0.009**	0.28 (0.09–0.83)	**0.022**
Gender (male = 1)	0.87 (0.39–1.93)	0.723		
Age	1.01 (0.97–1.04)	0.690		
T category (T1/T2 = 1)	5.25 (0.65–42.40)	0.119		
*N* category (*N*x/*N*0 = 1)	2.29 (0.52–10.04)	0.273		
Tumour grading (G1/G2 = 1)	3.29 (1.41–7.70)	**0.006**	2.50 (1.06–5.89)	**0.037**
Microvascular invasion (M0 = 1)	1.74 (0.65–4.68)	0.270		
Maximum tumour diameter	1.09 (0.95–1.26)	0.210		
Histologic classification (small duct = 1)	2.15 (0.94–4.93)	0.071		
TME pattern (desertic =1)	—	—		
Excluded	1.12 (0.47–2.67)	0.799		
Dispersed	0.91 (0.20–4.10)	0.905		
Structured	0.38 (0.05–2.90)	0.348		
CD8+	1.00 (1.00–1.00)	0.425		
TLS	0.93 (0.74–1.18)	0.566		
CD20+	0.963 (0.91–1.02)	0.198		

*Note*: Desertic: with an absence of T‐cell and B‐cell immune infiltration; Excluded: in which immune cells are present in the tumour margins but cannot penetrate the tumour bed; Dispersed: in which immune cells infiltrate throughout the tumour but cannot form organized networks; Structured: when immune cells form TLS that orchestrate the activation, maturation and intra‐tumoural distribution of immune cells. Bold indicates *p* < 0.05.

Abbreviations: HEV, high endothelial venule; HR, hazard ratio; TLS, tertiary lymphoid structures; TME, tumour microenvironment.

**TABLE 3:** Univariate and multivariate analysis of recurrence‐free survival.

Variables	Univariate analysis		Multivariate analysis	
	HR (95% CI)	*p*‐Value	HR (95% CI)	*p*‐Value
HEV (Low = 1)	0.23 (0.08–0.67)	**0.007**	0.31 (0.10–0.96)	**0.042**
Gender (Male = 1)	0.91 (0.41–2.03)	0.815		
Age	1.01 (0.97–1.04)	0.779		
T category (T1/T2 = 1)	5.04 (0.62–41.06)	0.130		
*N* category (*N*0 = 1)	2.08 (0.48–9.07)	0.328		
Tumour grading (G1/G2 = 1)	3.17 (1.36–7.39)	**0.008**	2.67 (1.11–6.41)	**0.028**
Microvascular invasion (M0 = 1)	1.65 (0.61–4.41)	0.322		
Maximum tumour diameter	1.09 (0.95–1.26)	0.203		
Histologic classification (small duct = 1)	2.31 (1.01–5.33)	**0.049**	2.34 (0.99–5.51)	0.052
TME pattern (desertic = 1)	—	—		
Excluded	1.06 (0.44–2.54)	0.892		
Dispersed	0.88 (0.20–3.99)	0.873		
Structured	0.34 (0.04–2.59)	0.295		
CD8+	1.00 (1.00–1.00)	0.475		
TLS	0.93 (0.73–1.17)	0.514		
CD20+	0.96 (0.91–1.02)	0.185		

*Note*: Desertic: with an absence of T‐cell and B‐cell immune infiltration; Excluded: in which immune cells are present in the tumour margins but cannot penetrate the tumour bed; Dispersed: in which immune cells infiltrate throughout the tumour but cannot form organized networks; Structured: when immune cells form TLS that orchestrate the activation, maturation and intra‐tumoural distribution of immune cells. Bold indicates *p* < 0.05.

Abbreviations: HEV, high endothelial venule; HR, hazard ratio; TLS, tertiary lymphoid structures; TME, tumour microenvironment.

## 4 DISCUSSION

ICC, a rare and poorly studied biliary tract cancer, features poor prognosis when compared with other solid malignancies, especially in individuals who are unsuitable for resection surgery.^21^ Still gemcitabine combined with cisplatin remains the first‐line chemotherapy for unresectable cholangiocarcinoma.^22^ However, the survival benefit of palliative treatment is very limited, which also leads to adverse side effects compared with supportive care.^23^ Multiple factors are responsible for the declining success rate of ICC treatment, such as the limited understanding of TME. Consequently, for individuals with ICC, novel immune biomarkers are urgently required.

Numerous research reports have highlighted that EC are essentially involved in normal physiological functions as well as multiple important diseases such as cardiovascular diseases, chronic inflammatory diseases, and malignant tumours.^24^, ^25^, ^26^, ^27^, ^28^ Despite common functions among all vascular EC, there is a remarkable degree of structural and functional heterogeneity in the microvascular bed across different lengths of the vascular tree and organs. HEV is one of the most striking examples,^28^ which exists in secondary lymphoid organs such as lymph nodes. Through HEV, lymphocytes migrate very efficiently from the blood to secondary lymphoid organs on a large scale. It has been estimated that up to 5 × 10^6^ lymphocytes per second pass through HEV.^14^ Early studies on breast cancer reported HEV density to be correlated with improved survival outcomes.^20^ In addition, HEV was also correlated with prolonged PFS and OS duration in oral, pharyngeal, and laryngeal cancers.

Moreover, tumour regression was positively correlated with HEV density in individuals with cutaneous melanoma.^29^, ^30^ However, to date, the function of HEV in ICC has never been reported. Therefore, clarifying the impact of HEV on the immune microenvironment and the prognosis of individuals with ICC is essential.

In a study of breast cancer, it compares the transcriptome information of HEV and non‐HEV tumour vessels in clinical breast cancer specimens. Endothelium of each type of vessel was isolated from tumour sections by laser capture microdissection. RNA‐seq was used to detect differentially expressed genes in EC.^31^ The results showed that there were significantly different gene expression patterns between HEV and non‐HEV tumour vascular EC. In order to eliminate data errors caused by lymphocyte contamination, the expression profile of non‐EC cell types, which are mainly composed of lymphocytes, was also studied. It was confirmed that the proteins encoded by these three genes (TCF7, TCL1A, and CD3E) were not expressed in human lymph node HEVs, and these genes were removed from the final list as potential contaminants.^31^ Finally, we selected the statistically significant genes (CHST4, CCL21, CCL19, IL33, ICAM1, MADCAM1, TSPAN7, MEOX2, ANKRD53, and ZNF280C) with a 2‐fold difference in expression level as HEV markers for subsequent analysis. Among them, CHST4 and IL33 have been shown to play important roles in the formation, maintenance, and function of HEV.^32^, ^33^, ^34^, ^35^ In the current research, 10 HEV‐related genes were retrieved from the literature, and then 42 stable and specific HEV‐related genes were identified by multiple iterations of differential genes analysis. These were the key genes enriched in corresponding immune response‐related pathways. With the consensus clustering algorithm, the GSE89749 cohort was classified into two clusters with varied profiles of HEV gene expression, namely C1 (high‐HEV subtype) and C2 (low‐HEV subtype). A further analysis showed that high‐HEV patients showed an improved prognosis and a dominant immune‐related phenotype. The up‐regulated genes in the high‐HEV subtype were mainly enriched in immune‐related biological processes. In contrast, the low‐HEV subtype displayed the opposite trend. These outcomes revealed that HEV was considerably linked to immune activation in the tumour immune microenvironment.

The link between TILs and the immune microenvironment has been extensively studied in various solid malignancies, indicating the role of TILs in favourable prognostic outcomes in ICC.^36^ Currently, an increasing number of studies have shown that the favourable prognostic effect of HEV is associated with TILs infiltration, including cytotoxic T lymphocytes, central memory T lymphocytes, and B lymphocytes.^16^ Conversely, Tregs have been reported to inhibit HEV development.^37^, ^38^ In addition, a previous study revealed that high‐HEV tumour levels were associated with adaptive immunity and T‐cell toxicity.^20^ In the current report, TME's composition in both subtypes was analysed, and it was discovered that the activation degrees of plasma cells, naive B cells, DC cells, and CD8^+^ T cells increased significantly.

In contrast, the activation degrees of memory B cells, M0 macrophages, mast cells, and Treg cells decreased dramatically in individuals of the high‐HEV subtype. In addition, Naive B cells, plasma cells, DC activation, and CD8 T cells were significantly positively correlated with HEV ssGSEA scores. By comparison, memory B cells, M0 macrophages, mast cell activation, and Treg cells showed clear opposite trends in the correlation analysis. Nevertheless, the low‐HEV subtype showed the opposite trend. In recent years, tumour‐associated TLSs have attracted much attention.^39^ TLSs are privileged sites of local antigen presentation and lymphocyte differentiation and thus provide an important environment for cellular and humoral immune responses against tumours.^40^ Moreover, intra‐tumoral TLSs are linked to improved prognosis and response to immunotherapy in most solid tumours.^40^, ^41^ This research showed HEV ssGSEA scores were highly positively correlated with TLS ssGSEA scores (Spearman's test, ρ = 0.7517, *p* < 0.001). In a prognostic model, premising immune cell infiltration score was created, and HEV activation was considerably linked to OS in individuals with ICC, implying that HEV can serve as an independent factor to predict the prognosis of individuals with ICC. Collectively, these findings indicate that HEV represents an immunocompetent TME in which the cancer immune cycle is functionally activated.

Due to recent technological advances in next‐generation sequencing and imaging approaches, spatial transcriptomic RNA sequencing has emerged as a cutting‐edge technology to systematically measure gene expression levels at distinct spatial locations in a tissue.^42^ To highlight the colocalization correlation between HEV and TLS in ICC, the spatial transcriptome data of ICC were obtained and re‐analysed in this study. It was observed that HEV and TLS were less enriched in the tumour region and showed a strong spatial colocalization relationship.

By fitting the change curves of the six cell types, HEV, and TLS scores, it was discovered that the cluster structure which is linked to the matrix region shaped the change patterns of EC, fibroblasts, T cells, B cells, and even HEV and TLS. A research has shown that ICI efficacy is considerably correlated with intra‐tumoral HEV.^43^ Despite the lack of research on the involvement of HEV in ICI, TLSs, which are HEV‐rich in ectopic lymphoid structures, were found to be good biomarkers for ICI therapy.^44^, ^45^ In this study, the immune checkpoint expression level was evaluated in two HEV subtypes. The results proved that most of these checkpoints were up‐regulated in the high‐HEV subtype. The TIDE algorithm was utilized to predict the response of patients to immunotherapy, and it was found that individuals with high‐HEV ICC benefitted more from immune checkpoint inhibitors. The association between HEV and immunotherapy outcomes was studied using the IMvigor210 cohort, and OS was also improved in high‐HEV patients. Though, HEV is not always linked to organized lymphoid structures, which is usually present in loose lymphoid aggregates. Consequently, an additional translational and clinical research is required to assess the potential of HEV as a predictive biomarker for immunotherapy in ICC. Further researches on the status of ICC mutations will help clinicians select the best immunotherapy technique. To illustrate, the latest research suggests that metastatic uveal melanoma may respond to MEK inhibitors in GANQ11‐driven melanoma.^46^ The variations in tumour intrinsic genomic alterations, TMB levels, co‐occurrence, and mutual exclusion were observed among frequently mutated genes in the TCGA‐CHOL ICC cohort. The findings of this research revealed a considerable variation between the high‐HEV and low‐HEV groups. In general, the high‐HEV group had a significant mutation rate and a lower TMB level. Especially, the ssGSEA score of the EP400‐mutation group was remarkably higher in comparison with the wild‐type group. EP400 is an ATP‐dependent chromatin remodelling enzyme that regulates DNA double‐strand break repair and transcription.^42^ It has shown that the N‐terminal domain of EP400 can increase the efficacy of chemotherapeutic drugs on cancer cells.^47^

Subsequently, immunohistochemical (IHC) staining of specimens from 95 individuals with ICC was performed, and it was noted that individuals with ICC in the high‐HEV group had more infiltration of CD8+, TLS, and CD20+ immune cells than those in the low‐HEV group. Representative samples with high‐HEV expression were selected for mIF staining in order to find the correlation between HEV and immune cells. The results showed an obvious colocalization relationship between HEV and immune cells. Meanwhile, individuals with ICC in the high‐HEV group had smaller tumour diameters, along with better disease‐free survival and OS. Traditionally, ICC can result from small intrahepatic bile ducts in the liver (septal and interlobular BD) as well as large intrahepatic bile ducts in the liver (segmental and regional BD).^48^ It has been reported that patients with small bile duct types have remarkably elevated survival rates in comparison to those with bold casts, as exemplified by Cholangiolocellular carcinoma.^49^, ^50^ Consistently, our study observed more HEVs in samples classified as small bile duct type. Recently, a study reported that TME should be considered as a whole, in which both its cellular composition and spatial organization should be considered.^51^ The TME of tumours can be divided into four types: ‘Desert’, ‘Excluded’, ‘Dispersed’, and ‘Structured’, with distinct immune infiltration levels.^51^ Consistent with our results, the high‐HEV group was substantially associated with subtypes rich in immune cell infiltration. KM analysis of 95 ICC patients also showed that HEV status significantly influenced survival outcomes. The high‐HEV patients had considerably longer RFS and OS than the individuals with low HEV. Finally, we also confirmed that HEV was an independent predictor of improved OS and DFS in univariate and multivariate Cox regression analyses.

For the very first time, this study demonstrated that high HEV expression was usually accompanied by more immune cell infiltration in individuals with ICC, and HEV was identified as an important immunological prognostic factor for RFS and OS in individuals with ICC who could undergo resection surgery. However, this research also has a few limitations. The cohort in this study features a limited sample size, consisting of only 95 individuals with surgically resected ICC. By using an external validation cohort, this research could be validated and strengthened in a larger sample size. Moreover, given the limited time and funding, merely the relationship between HEV and CD8+ TLS, CD20+ immune cells or structures was observed, whilst the specific mechanism of HEV effect on the immune microenvironment of ICC has not identified. These issues will be included in the next research.

## 5 CONCLUSIONS

This study for the first time demonstrated that high‐HEV expression was usually accompanied by more immune cell infiltration in individuals with ICC, and identified HEV as an important immunological prognostic factor for RFS and OS in individuals with ICC who could undergo resection surgery.

## AUTHOR CONTRIBUTIONS

Jun Chen, Chunping Jiang, Xiaohong Pu, and Yan Zhou conceived the project and designed the study. Yan Zhou wrote the article. Yan Zhou, Linxi Zhu, Shuo Zhang, and Xiaohong Pu performed the experiments. Qian Gu and Hongyan Wu analysed the clinical data. Jun Chen, Chunping Jiang and Xiaohong Pu obtained funding.

## FUNDING INFORMATION

This research was supported by funding for Clinical Trials from the Affiliated Drum Tower Hospital, Medical School of Nanjing University (2022‐LCYJ‐MS‐27); Major Project of Nanjing Health Science and Technology Development (ZDX22001); the Chinese National Science Foundation (82172631), and The Nanjing Municipal Administration of Health and Human Services (YKK21074); Research Project of Jinan Microecological Biomedicine Shandong Laboratory (JNL202204A, JNL202219B).

## CONFLICT OF INTEREST STATEMENT

The authors declare that they have no conflict of interest.

## Supporting information


**FIGURE S1.** Tumour microenvironments patterns of different HEV expression. (A) Representative images of different tumour microenvironments. (B) HEV expression in different tumour microenvironment (proportion).

## Data Availability

All the data obtained and analysed during our research can be provided by the corresponding author upon reasonable request.
