# Supplementary figures and images for: High endothelial venule is a prognostic immune‐related biomarker in patients with resected intrahepatic cholangiocarcinoma
Source: Cell Prolif. 2023 Jul 3;56(12):e13513. doi: 10.1111/cpr.13513 (PMC10693183; doi:10.1111/cpr.13513)

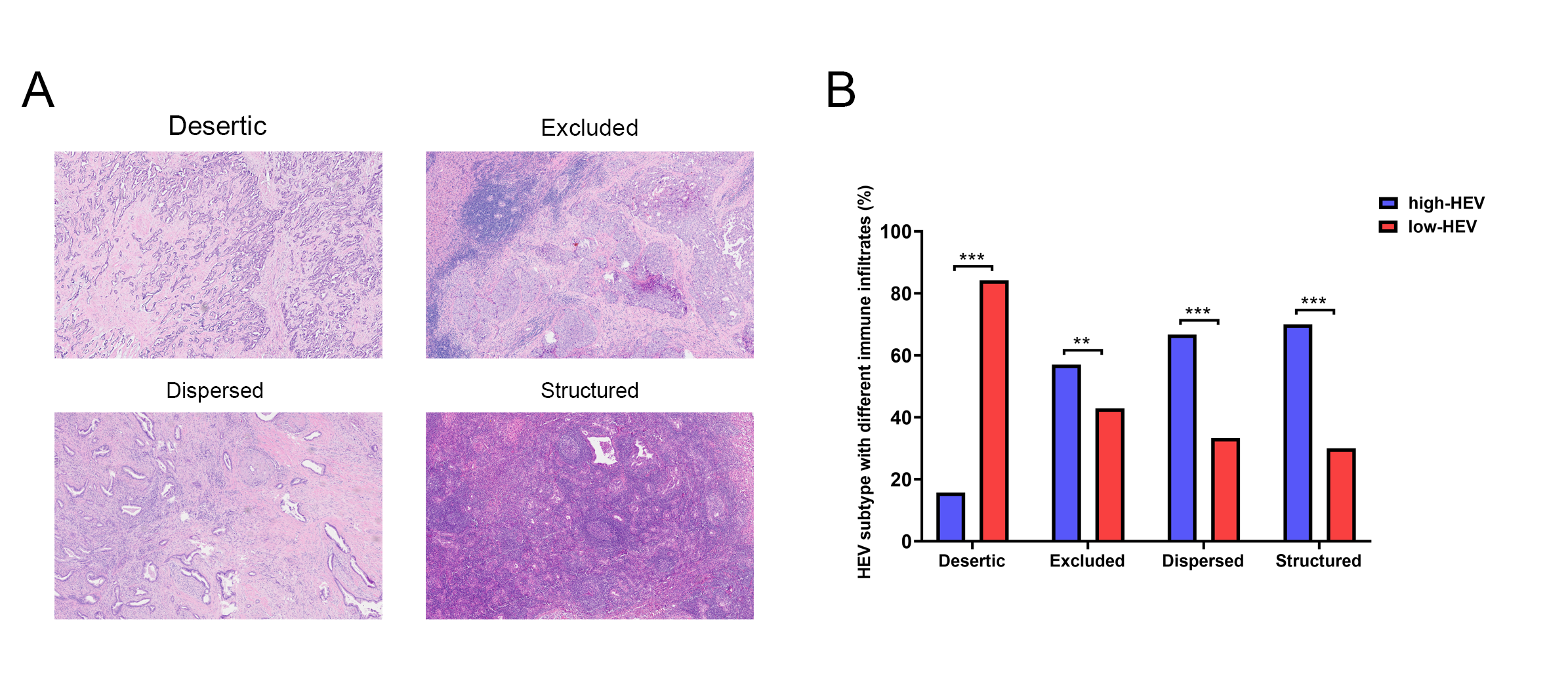

Supplement: Supplementary file 1 — FIGURE S1. Tumour microenvironments patterns of different HEV expression. (A) Representative images of different tumour microenvironments. (B) HEV expression in different tumour microenvironment (proportion). [file CPR-56-e13513-s001.tif]
